# Mandibuloacral dysplasia type A-associated progeria caused by homozygous LMNA mutation in a family from Southern China

**DOI:** 10.1186/1471-2431-14-256

**Published:** 2014-10-07

**Authors:** Di-Qing Luo, Xiao-Zhu Wang, Yan Meng, Ding-Yang He, Ying-Ming Chen, Zhi-Yong Ke, Ming Yan, Yu Huang, Da-Fang Chen

**Affiliations:** Department of Dermatology, The Eastern Hospital of The First Affiliated Hospital, Sun Yat-sen University, Guangzhou, 510700 China; Department of Epidemiology and Biostatistics, Peking University Health Science Center, Beijing, China; Department of Medical genetics, Peking University Health Science Center, Beijing, China; Department of Paediatrics, The General Hospital of People’s Liberation Army, Beijing, China; Department of Radiology, The Eastern Hospital of The First Affiliated Hospital, Sun Yat-sen University, Guangzhou, 510700 China; Department of Paediatrics, The Eastern Hospital of The First Affiliated Hospital, Sun Yat-sen University, Guangzhou, 510700 China

**Keywords:** Differential diagnosis, *LMNA* gene, Mandibuloacral dysplasia type A, Mutation, Progeria syndrome

## Abstract

**Background:**

Mandibuloacral dysplasia type A (MADA) is a rare autosomal recessive disorder, characterized by growth retardation, skeletal abnormality with progressive osteolysis of the distal phalanges and clavicles, craniofacial anomalies with mandibular hypoplasia, lipodystrophy and mottled cutaneous pigmentation. Some patients may show progeroid features. MADA with partial lipodystrophy, more marked acral, can be caused by homozygous or compound heterozygous mutation in the gene encoding lamin A and lamin C (*LMNA*). MADA and Hutchinson-Gilford progeria syndrome are caused by the same gene and may represent a single disorder with varying degrees of severity. MAD patients characterized by generalized lipodystrophy (type B) affecting the face as well as extremities and severe progressive glomerulopathy present heterozygous compound mutations in the *ZMPSTE24* gene.

**Cases presentations:**

We described a rare pedigree from Southern China, among them all three children presented with phenotypes of MADA associated progeria. The two elder sisters had developed severe mandibular hypoplasia associated progeria since the age of 1year. The eldest sister showed a progressive osteolysis. The youngest son of 10 months showed severer lesions than those of his sisters at the same age, and presented possible muscle damage, and his symptoms progressed gradually. Three genes mutations including *LMNA*, *ZMPSTE24* and *BANF1* were tested in the family. *LMNA* gene sequencing revealed a homozygous missense mutation, c.1579C > T, p.R527C for all three siblings, and heterozygous mutations for their parents, whereas no mutations of *ZMPSTE24* and *BANF1* genes was detected among them.

**Conclusions:**

The same homozygous mutation of c.1579C > T of *LMNA* gene led to MADA associated progeria for the present family. The course of osteolysis for MADA is progressive.

## Background

Mandibuloacral dysplasia type A (MADA [OMIM 248370]), is a rare autosomal recessive disorder, characterized by growth retardation, skeletal abnormality with progressive osteolysis of the distal phalanges and clavicles, craniofacial anomalies with mandibular hypoplasia, delayed closure of cranial sutures, clavicular hypoplasia, joint contractures, lipodystrophy, and pigmentary skin changes [1–3]. Some patients may show progeroid features. MADA with partial lipodystrophy, more marked acral, can be caused by homozygous or compound heterozygous mutation in the gene encoding lamin A and lamin C (*LMNA*). MADA and Hutchinson-Gilford progeria syndrome (HGPS) are caused by the same gene and may represent a single disorder with varying degrees of severity [1–3]. The severity of these features increases with the development of the patients. There are two patterns of lipodystrophy for MAD: type A (MADA) and type B (MADB). Type A, caused by the mutation of *LMNA* gene, is characterized by partial loss of fat from extremities with normal or excessive deposition in the face and neck [3, 4]; and type B, caused by the mutation in the zinc metalloproteinase (*ZMPSTE24*) gene, is characterized by generalized loss of subcutaneous fat affecting the face as well as extremities [3–5]. MADB patients can present severe progressive glomerulopathy. Some patients may develop metabolic complications such as impaired glucose tolerance, and hyperlipidemia due to insulin resistance and hyperinsulinemia [3]. Growth retardation and short adult height are the common presentations of MAD. Some patients may show premature aging features including bird-like facies, high-pitched voice, alopecia, skin atrophy, and nail dysplasia [2, 6].

Aging is a very complex question which perplexed scientists for many years, and its molecular basis and pathogenesis remain unknown. Progeria syndromes are rare disorders that involve premature aging and growth retardation which are genetically and phenotypically heterogeneous. Due to different molecular basis, there are two major types of progeria syndromes. One group depends on defects in helicase proteins which are responsible for DNA recombination and repair proteins, such as Cockayne syndrome (CSA and CSB) et al. [7, 8], which is also known as segmental syndrome. Another group is associated with defect of nuclear envelope proteins, encoded by *LMNA, ZMPSTE24* and *BANF1* et al., such as HGPS and MADA mutated in *LMNA*, MADB mutated in *ZMPSTE24*, and Nestor–Guillermo progeria syndrome (NGPS) in *BANF1* [4, 9, 10].

There are two genes reported to be responsible for MAD: *LMNA* [4] and *ZMPSTE24* [4, 5]. *LMNA* gene encodes integral nuclear lamina proteins. Due to alternatively splicing, there are two transcript isoforms: lamin A and lamin C [4], belonging to the intermediate filament family. The mutations of *LMNA* gene cause at least eight types of inherited disorders, including muscular, neurogenic, adiposocytopathies and progeria syndromes, such as Emery–Dreifuss muscular dystrophy type 2 [11], limb girdle muscular dystrophy type 1B [12], dilated cardiomyopathy type 1A [13], Charcot–Marie–Tooth disease type 2B1 [14], Dunnigan-type familial partial lipodystrophy [15], MAD, HGPS and restrictive dermopathy (RD) [16]. Another gene is *ZMPSTE24*, encoding a protease involved in posttranslational proteolytic processing of prelamin A to lamin A which is the mature form [5]. Compared to MADA patients with *LMNA* mutations, MADB patients with *ZMPSTE24* mutations have distinguished features including more severity of clinical phenotypes, early onset, premature birth, renal disease, calcified skin nodules and lack of acanthosis nigricans [5, 17].

We described a pedigree from Southern China, among them all three siblings presented with phenotype of MAD associated progeria and lipodsystophy, with the same homozygous mutation in *LMNA* gene mimicking the case reported by Agarwal et al. [18], while their parents showed healthy appearance with heterozygous mutations.

## Cases presentations

### Patients data and methods

The studies were approved by the Institutional Review Boards of the First Affiliated Hospital, Sun Yat-sen University, China, and written informed consent for the patients obtained from their parents, and consent for the parents signed by themselves.

### Patients descriptions

**Figure 1:**
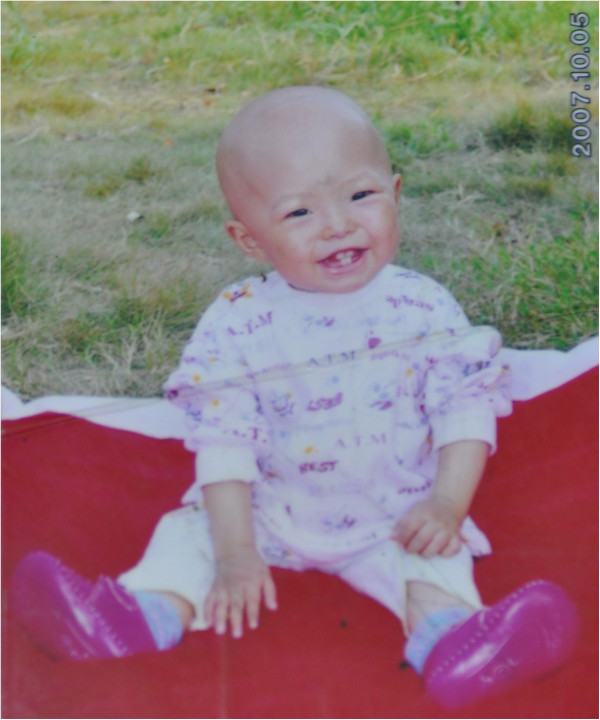
**Patient 1 showed decreased scalp hair growth at the age of 1 year.**

Patient 1, belonging to a non-consanguineous parents, was a 7-year old Han Chinese girl. She was born full-term with a birth weight of 2.4 kg and a length of 48 cm, and did not exhibit any abnormalities until the age of 10 months, when her upper limbs were noted with mottled hyperpigmentation, which progressed gradually associated with sclerodermatous change thereafter. At the age of 12 months, her parents noticed that she had swelling of hands and fingers with decreased mobility, and had decreased scalp hair growth (Figure 1), and failed to thrive. She had her first walk at the age of 14 months. At the age of 22 months, finger joints became painful and stiff. At the age of 26 months, similar mottled hyperpigmentation, thin skin and sclerodermatous change presented over the lower limbs and hips; and stiff feet joints were present; those made the girl have limited activity. All the symptoms progressed slowly. At her age of 30 months, based on the biopsy and laboratory test results, she was diagnosed as scleroderma, and was treated with prednisone 10 mg daily in combination with Chinese medicine, the steroid was then tapered gradually and had lasted for more than 2 years till the end of treatment. During the treatment, the cutaneous lesions progressed slowly associated with appearing of a bird-like face with beaked nose and bulbous cheeks, swallowing difficulties and thin skin, only the joint stiffness had mild improvement. Since the age of 5 years, carious and ragged teeth and hair alopecia including loss of eyebrow appeared. She also developed swallowing difficulty with frequent vomiting or choking, especially when drinking water. Defecating difficulty with frequent anal fissure were noted as well.

**Figure 2:**
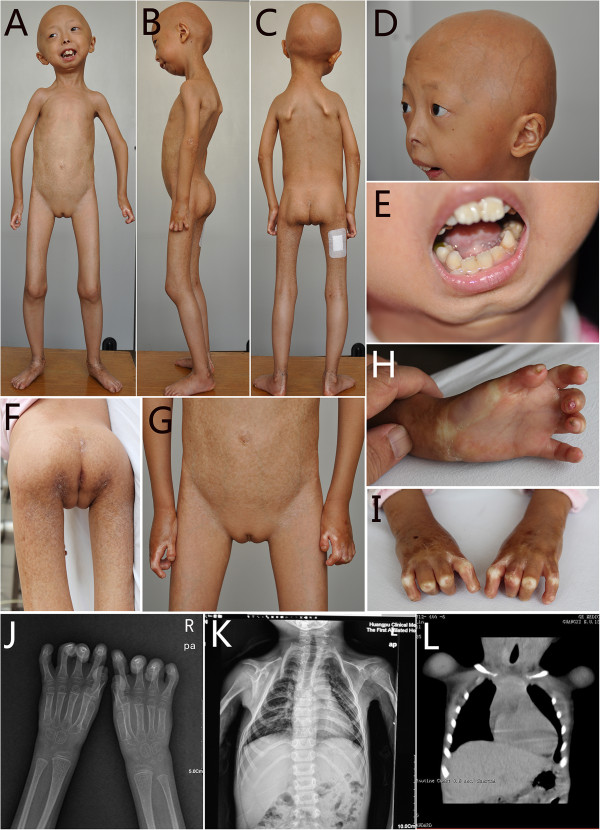
**Patient 1 shows diffuse scalp hair loss, decreased eyebrow, prominent scalp veins, bulbous cheeks, tapered nasal tip, irregular teeth and lower jaw dental crowding, loss of subcutaneous fat, and mottled pigmentation and sclerodermatous changes over her trunk and lower limbs with protuberant abdomen and easily visible veins on abdomen (A-G), thickened skin on heels and around the ankles (A-C), severe contractures at the interphalangeal joints of the hands with flexion deformity of fingers (H, I).** Lower skeletal density in upper limb with flexion deformity of fingers, and delayed bone age with absence of the lesser multangular bone, the great multangular bone, and the scaphoid bone **(J)**. Pyriform thorax with absence of the clavicles, and absence of posterior parts of the 2^nd^ and 3^rd^ ribs on both sides, and thin posterior parts of the 4^th^ to 6^th^ ribs on left side and the 4^th^ on right **(K)**. Normal clavicle images on the prior chest scan of computed tomography in 2009 **(L)**.

On examination, her weight (9 kg), height (95 cm), and head circumference (45.5 cm) were below the normal range (<mean-3 standard deviation (SD), all were below the third centile). She showed an extremely short stature, and distinctive face with diffuse scalp hair loss and decreased eyebrow, prominent scalp veins, bulbous cheeks, tapered nasal tip, irregular teeth and lower jaw dental crowding, and mandible hypoplasia (Figure 2A-E). She had loss of subcutaneous fat over the entire body, and mottled pigmentation and sclerodermatous changes over her trunk and lower limbs with protuberant abdomen and easily visible veins on abdomen (Figure 2F-G). She had thickened skin on heels and around the ankles (Figure 2A-C), and had a varus deformity of the knees with wide-based gait and shuffling. Severe contractures at the interphalangeal joints and the hands with marked finger tip rounding and nail atrophy had resulted in flexion deformity of fingers, and decreased mobility (Figure 2H, I). Mild elbow and knee contractures were also observed, but no spine rigidity was present. She had mild weakness of neck muscles with somewhat dropping head. She was absent for circumoral cyanosis. Her mental development including calculation was normal.

Complete hemogram, erythrocyte sedimentation rate, urine examination, liver and renal function tests, serum glucose and phosphocreatine kinase were within normal limit. The serum CK-MB was 36 U/L (normal range: 0 ~ 25 U/L). The serum lipid profiles showed a decrease in high density lipoprotein cholesterol (0.93 mmol/L, normal range: 1.09-1.63 mmol/L) with the other parameters being normal. X-ray findings of extremities showed lower skeletal density, flexion deformity of fingers, and delayed bone age with absence of the lesser multangular bone, the great multangular bone, and the scaphoid bone (Figure 2J). Radiological changes in chest revealed pyriform thorax, absence of clavicles, and absence of posterior parts of the 2^nd^ and 3^rd^ ribs on both sides, and thin posterior parts of the 4^th^ to 6^th^ ribs on left side and the 4^th^ on right (Figure 2K). However, the chest scan of computed tomography showed normal clavicles 4 years ago (Figure 2L). B-ultrasonic showed loss of the subcutaneous fat. Electromyogram for the extremities showed normal conduction velocity and normal wave. Repeated electrocardiograms showed normal.

Patient 2, the younger sister of patient 1, was 3-year- and 3- month-old. She was delivered after an uneventful pregnancy at 34 weeks of gestation (length: 45 cm; weight: 1.8 kg). She showed normal motor and mental development since the birth, and the growth was regular until she was noted sequently to have growth retardation, mottled hyperpigmentation, cutaneous sclerodermatous change, thin skin, decreased subcutaneous fat and joint stiffness at the age of 12 months. The symptoms developed gradually, but her parents noticed that all her symptoms progressed more rapidly than her older sister’s. Thereafter, progressive loss of hair and eyebrows, swallowing difficulty and bulbous cheeks were present slowly. She also complained that water-drinking may make her choke with ease. She had her first walk at the age of 18 months.

**Figure 3:**
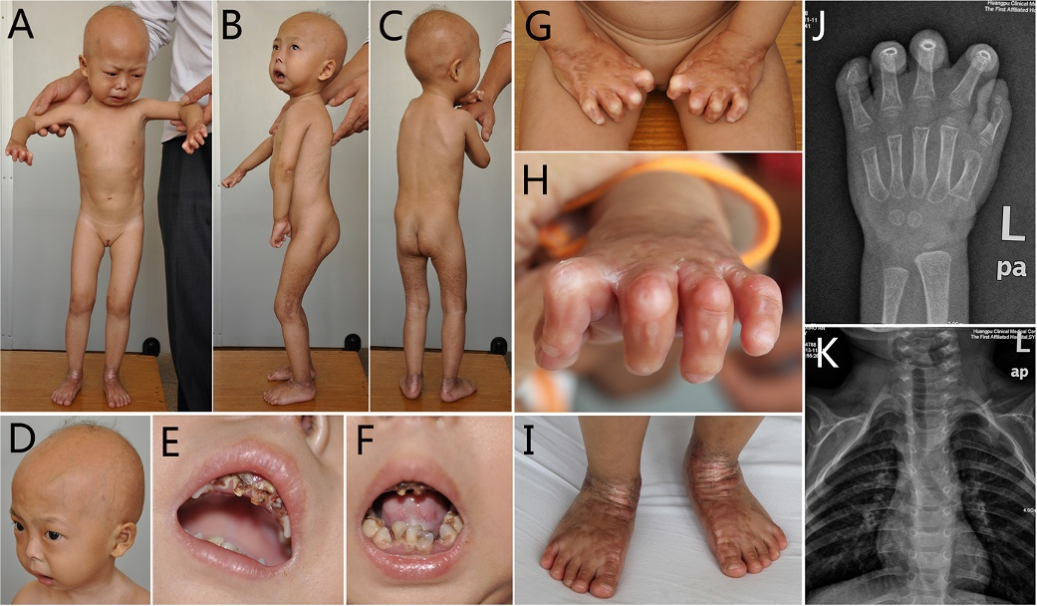
**Patient 2 shows a senile appearance with hyper- or hypo-pigmentation, sclerodermatous changes and thin skin on the lower abdomen, buttocks, elbows and the lower extremities, lipodystrophy of the gluteal region and extremities (A-C); sparse scalp hair with easily visible veins (D); irregular and carious teeth and crowded teeth on the mandible (E,F); severe contractures at the interphalangeal joints and the hands with abnormal posture (G,H), and coarse and thickened skin around the ankles and on the back of feet in symmetry (I).** X-ray findings of hand show flexion deformity of fingers of hands and delayed bone age **(J)**. Radiological changes in chest reveal pyriform thorax , absence of right clavicle, and absence of mid-lateral part with thin interior part of left clavicle, and thin posterior parts of the 2^nd^ and 3^rd^ ribs on both sides with disconnection of posterior parts of the 3^rd^ rib on left side and the 2^nd^ and 3^rd^ on the right **(K)**.

The examination showed her weight (7.5 kg), length (79 cm) and head circumference (44.3 cm) were below the normal range (<mean-3SD, below the third centile), with a senile appearance (Figure 3A-C). She had hyper- or hypo-pigmentation, sclerodermatous changes and thin skin on the lower abdomen, buttocks, elbows and the lower extremities (Figure 3A-C). Sparse scalp hair with easily visible veins was present (Figure 3D). Irregular and carious teeth, and crowded teeth on the mandible were also observed (Figure 3E,F). Lipodystrophy on gluteal region, extremities (Figure 3B,C) and palmoplantar areas was noted. Severe contractures at the interphalangeal joints and the hands with marked finger tip rounding had resulted in abnormal posture and decreased mobility (Figure 3G,H). Her nails were mildly dystrophic. There was coarse and thickened skin on the back of hands, around the ankles and on the heels in symmetry (Figure 3I). Her gait was waddling. No circumoral cyanosis was present. Her mental development was normal. Repeated electrocardiograms showed normal.

The laboratory tests including biochemistry (including serum phosphocreatine kinase) and lipid profiles were normal or within normal limit, except an increase of serum CK-MB (56 U/L, normal range: 0 ~ 25 U/L) and a decrease of high density lipoprotein cholesterol (0.68 mmol/L, normal range: 1.09-1.63 mmol/L). X-ray findings of extremities showed flexion deformity of fingers of hands and delayed bone age (Figure 3J). Radiological changes in chest revealed pyriform thorax as her elder-sister presented, absence of right clavicle, and absence of mid-lateral part with thin interior part of left clavicle, and thin posterior parts of the 2^nd^ and 3^rd^ ribs on both sides with disconnection of posterior parts of the 3^rd^ rib on left side and the 2^nd^ and 3^rd^ on the right (Figure 3K). B-ultrasonic showed the thickness of subcutaneous fat was 4 mm on the involved areas of both thighs. Electromyogram for the extremities showed possibility of myogenic damage.

Patient 3, the youngest sibling in the pedigree, was a 10-month-old boy. He was also delivered after an uneventful pregnancy at 37 weeks of gestation (length: 48 cm; weight: 3.2 kg). He was noticed hypermyotonia and swelling of lower extremities at the age of 8 months with gradual progression. His parents noticed that his lesions were more severe and progressed more rapidly than both sisters did. His motor and mental were normal at the time.

**Figure 4:**
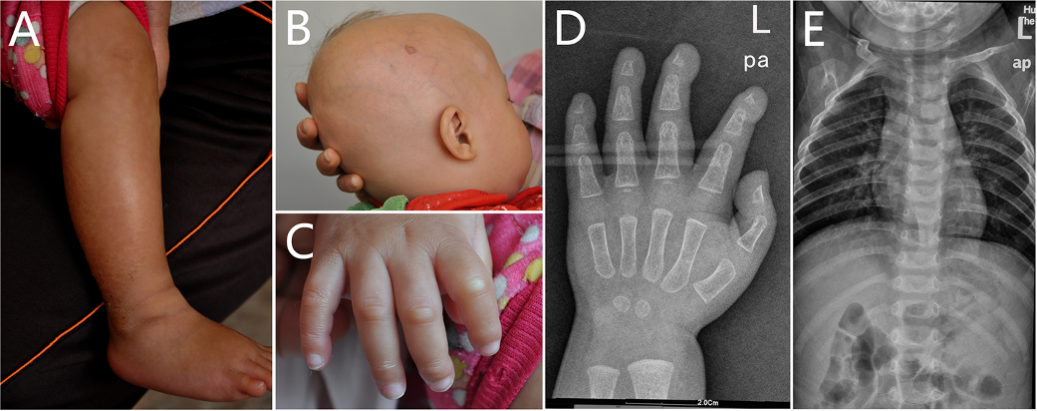
**Patient 3 shows swelling with mild hyperpigmentation on lower limb (A), decreased scalp hair with prominent scalp vein (B) and mild contractures at the interphalangeal joints of hands (C).** X-ray findings show acro-osteolysis of distal phalanges, normal clavicles and ribs **(D, E)**.

Examination showed his weight (7.7 kg) and height (68 cm) were below the normal range (<mean-2SD, below the third centile), and the head circumference (44.5 cm) was in normal range (equal to the twenty centile). His lower extremities were found swelling with mild hyperpigmentation (Figure 4A), and increase of muscular tension. Decreased scalp hair with prominent scalp vein (Figure 4B) and mild contractures at the interphalangeal joints of hands were also noted (Figure 4C). No other abnormalities including circumoral cyanosis were observed at the time. The laboratory tests including biochemistry and lipid profiles were normal or within normal limit, except increase of lower-density lipoprotein cholesterol (4.00 mmol/L, normal range: 1.94-3.61 mmol/L), serum CK (579 U/L, normal range: 250–200 U/L) and CK-MB (80 U/L, normal range: 0 ~ 25 U/L). The parents refused to take muscle biopsy. X-ray findings of the hands showed acro-osteolysis and thorax X-ray including clavicle and ribs showed normal (Figure 4D,E). Thickness of subcutaneous fat was 11 mm on the involved areas of both thighs. Electromyogram for the extremities showed possibility of myogenic damage.

In a phone follow-up during the manuscript was being revised (the patient was 1-year and 10-month old at the time), his mother reported that his mental was still normal, and his first walk began at his age of 14 months; all his symptoms developed more rapidly than his elder sisters did, and mildly progressive contractures of knee joints appeared recently which made him have limited activity.

Their mother was a 42-year-old Han Chinese woman with healthy appearance (length: 152 cm, weight: 50 kg). Her radiological changes in chest and laboratory tests including biochemistry and lipid profiles were normal or within limit, except for increased lower-density lipoprotein cholesterol (4.06 mmol/L, normal range: 1.94-3.61 mmol/L). She had no abortion. Their father was a 41-year-old Han Chinese man with normal appearance (length: 170 cm; weight: 70 kg). His chest x-ray and laboratory tests were also normal or within normal limit, except decreased high-density lipoprotein cholesterol concentration of 0.80 mmol/L and increased lower-density lipoprotein cholesterol concentration of 4.19 mmol/L. Both fasting glucose and 2-hour postprandial blood glucose of the parents were normal.

### Molecular analysis of the *LMNA, ZMPSTE24*and *BANF1*genes

Genomic DNA was isolated from peripheral blood with a DNA isolated kit (Aidelai, CN)) according to the manufacturer’s protocol. Direct sequencing of the entire coding region and the surrounding intron-exon boundaries of the *LMNA, ZMPSTE24* and *BANF1* genes were conducted in the proband (the oldest sister of this pedigree). Primers of *LMNA ZMPSTE24* and *BANF1* genes were designed by primer3.0 software. The PCR reaction was assembled in a 25 μl reaction volume, containing 50 ng genomic DNA, 5 pmol of each primer, 1x Taq mix (Aidelai, CN). PCR was conducted on ABI 9800 using the touchdown cycle protocol modified as followed by a 3-step cycle (95°C, 5 min, 95°C, 45 s, 59°C, 45 s, 72°C, 45 s, 2cylces, 95°C, 45 s, 57°C, 45 s, 72°C, 45 s, 2cylces ; 95°C, 45 s, 55°C, 45 s, 72°C, 45 s, 2cylces; 95°C, 45 s, 53°C, 45 s, 72°C, 45 s, 30cylces; 72°C, 10 min). The PCR product was purified to remove primers and dNTPs and sequenced using ABI Prism 3100 (Perkin-Elmer Applied Biosystems, Foster City, CA). Sequence of PCR products was analyzed with Chromas 2.22.

## Results

**Figure 5:**
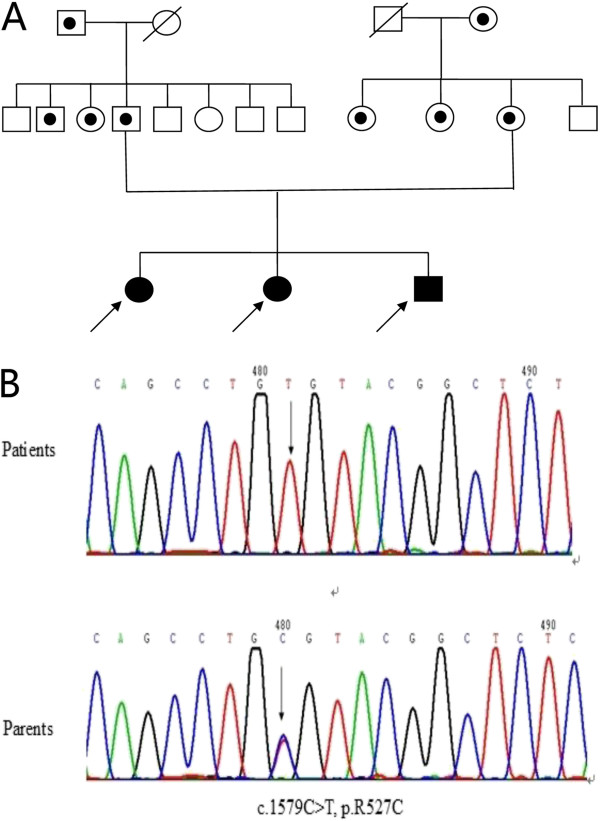
**Pedigrees of the patients with MADA.** Affected individuals are shown as *filled black symbols*, whereas heterozygous subjects are shown as *a dot inside* **(A)** *.* The results of sequencing LMNA gene exon9 of the affected siblings and heterozygous parents show a homozygous mutation p R527C in all of three affected siblings **(B)**.

Screening of the exons and the adjacent introns and splice sites revealed no disease causing variants in *ZMPSTE24* and *BANF1* genes in the probands of this pedigree. All three siblings were homozygous for the same mutation of c.1579C > T in exon9 of *LMNA*, which resulted in the mutation of p.R527C (Figure 5). The mutation segregated in an autosomal recessive inherited manner in the pedigree. Their parents, grandpa and grandma-in-law, their father’s second brother and the elder-sister, and both of their mother’s sisters were carriers.

## Conclusions

The present pedigree showed severe phenotype of MADA associated with progeria syndrome in all of three siblings. Patient 1 exhibited typical phenotype of MADA, showing the initial symptoms in the first year of life, and postnatal growth retardation, hair loss, joint stiffness, lipodystrophy, limited mobility, mandibular and clavicular hypoplasia, and clavicle and rib osteolysis. The X-Ray results about clavicular changes of three siblings showed that osteolysis in this pedigree may progress gradually, especially patient 1 whose clavicles were present in early stage and disappeared later, suggesting the course of osteolysis is chronic. The present patients must be distinguished from HGPS, which is also characterized by aging appearance, early onset, and rapid progression [9]. It is considered that the phenotypes of both syndromes have overlap, but circumoral cyanosis and vascular complications which usually are the main causes of death for HGPS seem to be the peculiar features in HGPS [9, 18]. Our patients were absent for vascular complications such as myocardial infarction, intracranial bleeding, stroke and circumoral cyanosis, we considered they were MADA rather than HGPS. Besides severe mandibuloacral dysplasia with clavicle and rib hypoplasia, and delayed bone age, our patients showed typical features of progeria, such as alopecia, loss of eyebrows and eyelashes, bird-like nose, coarse and senile appearance. All these symptoms supported the diagnosis that the present pedigree was severe MADA associated with progeria syndrome. Of course, they were too young at the time to exclude the possibility developing vascular complications in future. Typical MADA is caused by the p.R527H mutation in the *LMNA* gene. Other mutations cause different phenotypes and in general are associated with more severe progeroid features similar or identical to HGPS. As the matter of fact, the differential diagnosis between MADA and HGPS in these last cases is not always easy because the gene is the same and these conditions may represent a single disorder with varying degrees of severity. As Holter monitoring is important in detecting cardiac arrhythmia, it is highly recommended in the patients with MADA, progeria and myopathy if possible.

Laboratory tests showed that the two older sisters had decrease of high density lipoprotein cholesterol, and patient 3 had increased low-density lipoprotein cholesterol, all these indicated metabolic disorder. Such conditions are considered the results of insulin resistance and diabetes in MADA patients [6], although all three siblings showed normal serum glucose at the time of testing.

All the siblings in present pedigree were detected with the same homozygous *LMNA* mutation (p.R527C), which mimicked the case reported by Agarwal et al. [18]. But the present patients had some features which were different from the prior report [18] including: first, all the present patients began their subtotal alopecia at the age of 1 year while the previous case had not mentioned; second, the present patients had more severe osteolysis including absence or part absence of clavicles and ribs, especially patient 1; third, possibility of muscle damage was present for patient 2 and 3, based on increased phosphocreatine kinase and abnormal electromyogram results. Muscle damage might indicate overlapping syndrome of MAD, atypical progeria and myopathy, similar as described by Kirschner, et al. [19].

The mutations of *LMNA* gene cause several types of inherited disorders, but the phenotypes are diverse. The Laminopathies are very complex, due to the multiple functions of lamin A and lamin C, such as maintenance of nuclear integrity, DNA replication and gene expression [20, 21]. There have been previously reported four mutation types in the code 527 of *LMNA* gene including R527H, R527C, R527P and R527L, which can cause different inherited disorders, respectively [4, 18, 22]. The homozygous R527H mutation in exon9 of the *LMNA* gene was reported commonly in patients with MADA [4, 6, 23]. The previous studies [18, 24] reported that R527H disrupts the bridge between Arg527 and Glu537 by predicting the three-dimension structure of the protein, and also observed similar salt bridge disruption when Cys527 was substituted for Arg. The present siblings had the same R527C mutation as Agarwal et al. [18] described, but their clinical phenotypes were different, we speculated that such conditions may result from different inherited backgrounds in different populations, but also may result from a variable expressivity of *LMNA* gene in the various cases. Although both clinical phenotypes and progressions of patient 1 and 2 were similar, but patient 3 had earlier onset (only 8 months old) with more progressive development, and presented atypical symptoms with possibility of muscle damage. The reasons for such atypical features we speculated may be the boy was in early stage, but we also can’t exclude whether there are some epigenetic factors or whether other genes influenced the pathogenesis of our patients. Of course more extensive tests and evidence are needed to support the speculation. The homozygous mutation R527C associated with HGPS has been recently reported in another two pedigrees from Southern China [25, 26], considering the respective frequencies of the diseases, such events for Southern Chinese population are just fortuitism or whether there is a founder effect or not needs further study.

### Accession numbers

The Genbank (http://www.ncbi.nlm.nih.gov/gquery/gquery.fcgi) accession number for the complete canine *LMNA* DNA sequence is NG_008692, The Genbank accession number for the *LMNA* mRNA sequence is NM_005572. The Genbank accession number for the *ZMPSTE24* DNA sequence is NG_008695, The Genbank accession number for the *BANF1* DNA sequence is NG_031874.

### Requesting consent statement

Written informed consent was obtained from the patient’s parents for publication of this case report and any accompanying images. A copy of the written consent is available for review by the Editor of this journal.

## Abbreviations

MAD: Mandibuloacral dysplasia
MADA: Mandibuloacral dysplasia type A
MADB: Mandibuloacral dysplasia type B
NGPS: Nestor–Guillermo progeria syndrome
HGPS: Hutchinson-Gilford progeria syndrome
RD: Restrictive dermopathy
SD: Standard deviation
ZMPSTE24: Zinc metalloproteinase.
